# Panic Buying in Bangladesh: An Exploration of Media Reports

**DOI:** 10.3389/fpsyt.2020.628393

**Published:** 2021-01-18

**Authors:** S. M. Yasir Arafat, Kum Fai Yuen, Vikas Menon, Sheikh Shoib, Araz Ramazan Ahmad

**Affiliations:** ^1^Department of Psychiatry, Enam Medical College and Hospital, Dhaka, Bangladesh; ^2^School of Civil and Environmental Engineering, Nanyang Technological University, Singapore, Singapore; ^3^Department of Psychiatry, Jawaharlal Institute of Postgraduate Medical Education and Research (JIPMER), Puducherry, India; ^4^Department of Psychiatry, Jawahar Lal Nehru Memorial Hospital (JLNMH), Srinagar, India; ^5^Department of Administration, College of Humanities, University of Raparin, Ranya, Iraq

**Keywords:** panic buying, Bangladesh, news reports, content analysis, COVID-19

## Abstract

**Background:** As an erratic human behavior, panic buying is an understudied research area. Although panic buying has been reported in the past, it has not been studied systematically in Bangladesh.

**Aim:** This study aimed to explore the characteristics of panic buying episodes in Bangladesh in comparison to current concepts.

**Methods:** A retrospective and explorative search were done using the search engine *Google* on November 6, 2020, with the search term “panic buying in Bangladesh.” All the available news reports published in the English language were extracted. A thorough content analysis was done focusing on the study objectives.

**Results:** From the initial search, a total of 30 reports were extracted. However, six reports were not included based upon the exclusion criteria, resulting in an analysis of 24 reports. Five panic buying episodes were identified, discussing the precipitating events, responsible factors, goods acquired through panic buying, and prevention measures. Flood, curfew, COVID-19, and export ban were found to be precipitating events. Media reports frequently mentioned prevention strategies, expert opinion, supply chain status, rationing, and government action. The reported goods that were panic bought were items necessary for daily living such as rice, oil, spices, and safety products such as hand sanitizer and masks.

**Conclusion:** The study revealed preliminary findings on panic buying in Bangladesh; however, they are aligned with the current concept of it. Further empirical studies are warranted to see the geographical variation, precise factors, and to test the culturally appropriate controlling measures.

## Introduction

Panic buying (PB) is an erratic human behavior that has been noticed in at least 93 countries all over the world during the COVID-19 pandemic (1). Outbreaks of infectious diseases can trigger feelings of uncertainty and violate an individual's sense of control (2, 3). Fueled by concerns about running out of certain items and goods, people tend to indulge in panic buying which gives them a semblance of control over a situation (1). Panic buying has been defined as “the phenomenon of a sudden increase in buying of one or more essential goods in excess of regular need provoked by adversity, usually a disaster or an outbreak resulting in an imbalance between supply and demand” (4). Several important aspects have been considered for PB, such as a sharp increase in the purchase of important necessary goods in excess of needs, usually precipitated by adverse events. It may be impulsive or well-planned (5).

**Figure 1:**
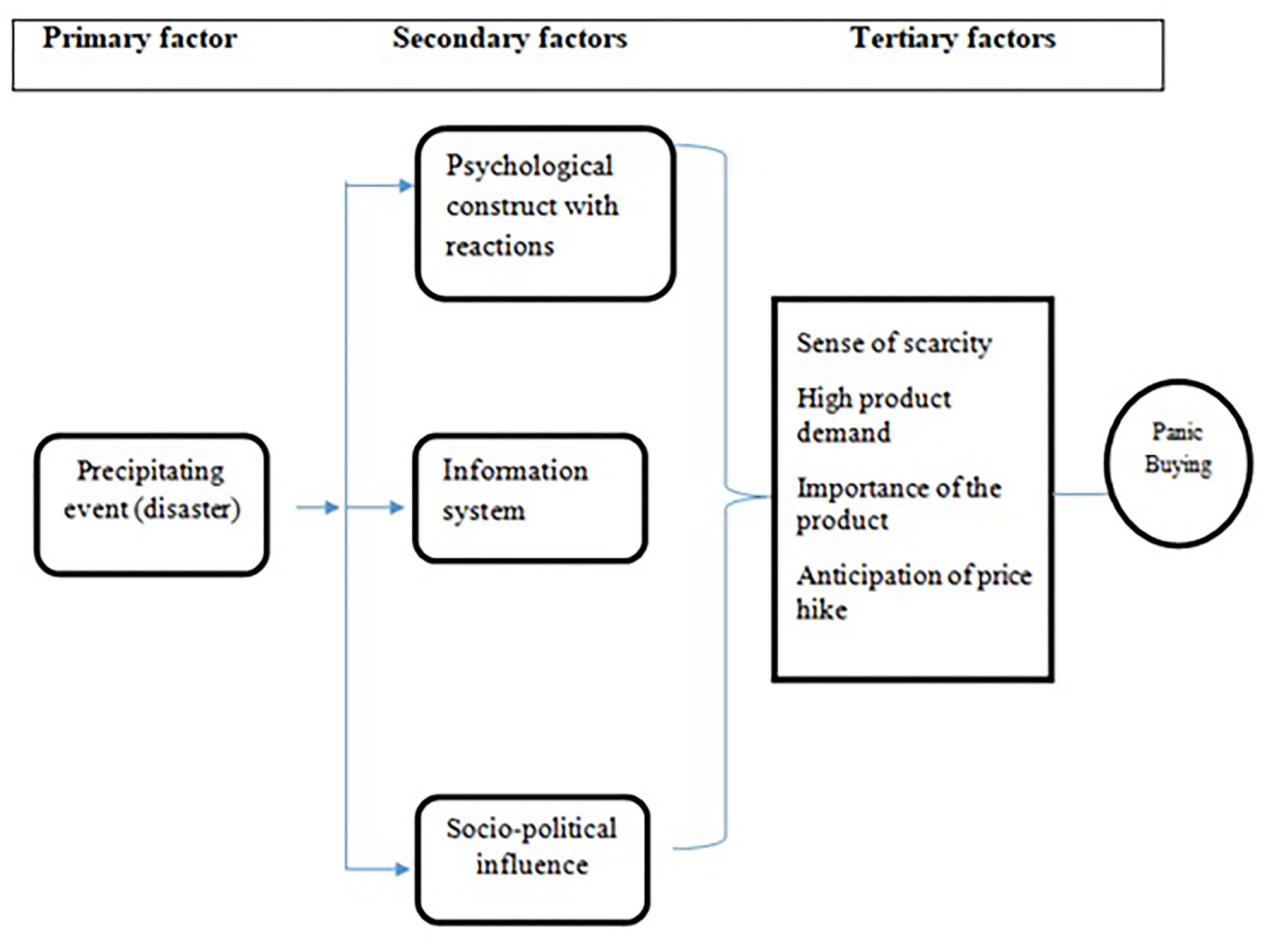
Responsible factors for panic buying [adapted from (1)].

A previous study also postulated a causative model of PB mentioning that any adverse precipitating event usually stimulates this behavior (1). As per the model, the adverse stimuli act as precipitating events and initiate the behavior (1). Subsequently, secondary factors (psychosocial construct and information system), and tertiary factors (increased demand, the necessity of the product, supply chain, and anticipation of price hike) interact and shape it (1) (Figure 1). Several psychological factors have been postulated such as perceived scarcity, gaining control, fear of uncertainty, media influence, social behavior explaining the PB (1, 2). One study identified that a perceived scarcity, increased demand, necessary goods, the anticipation of price hike, any adverse situations, rumors, psychological reactions, social influences, a lack of trust in authority (government action), and experience were the attributed factors of PB (1).

PB has many negative effects such as disruption of supply chains, artificial commodity shortages, and stoke price rise. Furthermore, large crowds and queues appearing in retail spaces and stores could result in further clusters. Some prevention strategies have been proposed such as responsible media reporting, kinship promotion, rationing, assurance from the authority, some psychological measures to prevent the episodes (6, 7).

Given this scenario, there is a need for further research into the precipitating factors of PB, evaluating the responsible factors, and possible mitigating strategies. Country specific research on PB is limited though, and it is crucial to consider prevention strategies. Against this background, we conducted the present analysis to assess the characteristics of panic buying episodes in Bangladesh, a populous country in South-East Asia. In particular, we aimed to evaluate precipitating events, responsible factors, goods of panic buying, and control strategies. Driven by previous research methods in PB, we used media reports to identify articles related to PB for analysis. The goal was to provide country-specific data that may inform management and prevention strategies to control PB.

## Materials and Methods

### Data Collection

**Figure 2:**
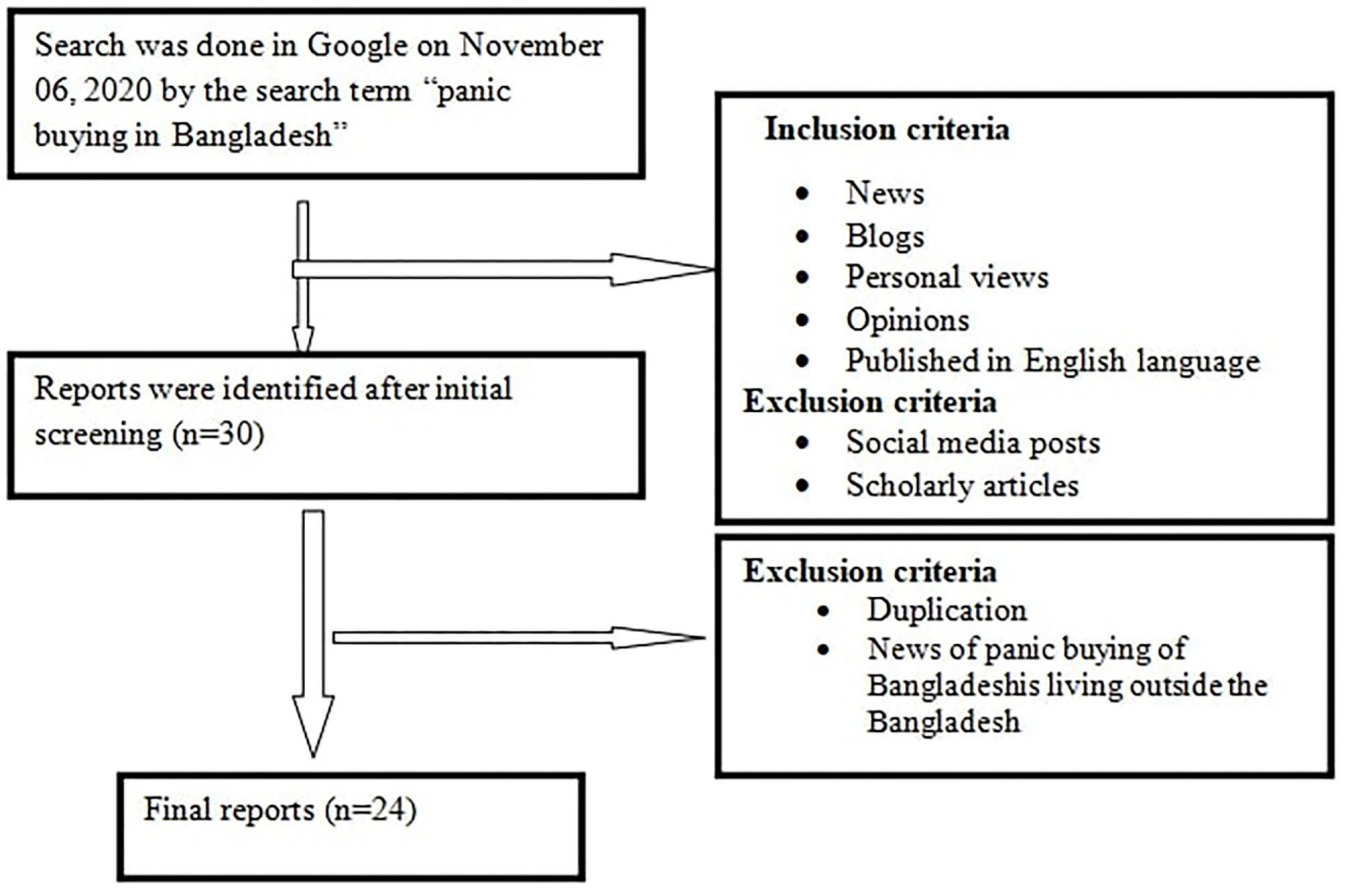
Flow chart showing study sample selection.

A retrospective and explorative search were done in Google on November 6, 2020, with the search term “panic buying in Bangladesh.” All the available news reports published in the English language were extracted. A thorough content analysis was done focusing on the study objectives. A method of three previous similar studies was followed to extract the reports, analyze the contents, identify the panic buying goods, assess the media reports, trace the responsible factors, and mention the controlling strategies (1, 4, 6). The detailed data extraction is outlined in Figure 2.

### Inclusion Criteria

The inclusion criteria for this analysis include reports from any report discussing panic buying from the media covering news, blogs, personal views, opinions that were published in English. Vernacular (Bangla) reports were not considered due to a lack of specification of the exact Bangla word for “panic buying.”

### Exclusion Criteria

Social media posts, scholarly articles, duplications, and news of the panic buying behaviors of Bangladeshis living outside Bangladesh were excluded from the study. Social media posts were excluded because they are supposed to be more emotionally charged and are very often biased. We excluded scholarly articles because we aimed to assess primary observations reported in the media. Various types of scholarly articles with various objectives would dislocate the research focus. Moreover, conformity bias, group-thinking, and herd behavior may be considered as potential sources of biases in social media posts (1).

### Statistical Analysis

We used the Microsoft Excel spreadsheet 2019 version for data coding. As a preliminary explorative study detail, statistical analysis was not performed.

### Ethical Statement

The study was conducted in compliance with the declaration of Helsinki (1964). As we analyzed publicly available media reports, no formal ethical approval was obtained.

## Results

From the initial extraction, a total of 30 reports were retrieved. However, six reports were dropped after considering the exclusion criteria, resulting in the analysis of 24 reports (Figure 1). From the 24 reports, five panic buying episodes were identified and the precipitating events, responsible factors, goods of panic buying, and prevention measures were discussed.

### Precipitating Events

From the reports, flood, curfew, COVID-19, and export bans (2) were found to be precipitating events that initiated the PB. All the reported episodes had a precipitating event like a flood, curfew, COVID-19, and export ban of onion (Table 1).

**Table 1:** Detail contents of reports (*n* = 24).

	**Date of publication**	**News outlet**	**Main theme**	**Responsible factors of PB**	**Circulation**	**Goods**	**Events**	**Prevention strategy**	**Precipitating event**
Panic buying pushes food prices up in Bangladesh (8)	Sep 11 1998	ReliefWeb	It reported a PB event and prevention activity	Rumor of flood	International	Rice, wheat, sugar, salt, onion, pulse, potatoes, spices	Increase demand and price, people buying extra amount	Assurance of stocks	Flood
Bangladesh imposes curfew after three days of student riots (9)	Aug 22 2007	The Guardian	It mentioned PB in response to the curfew	Curfew due to student riot	International				Curfew
Soaring onion prices create panic in Bangladesh (10)	Nov 22 2019	Global Voices	The report discussed the PB episode and government's action to prevent it	India banned the export of onions	International	Onion		Import from alternative sources, reduced use of onion	Export ban
Panic buying triggers mask, sanitiser crises (11)	Mar 9 2020	The Independent	It described an episode of PB	Detection of the first COVID-19 case	Local	Masks, sanitizers	Increase demand and price, stock out		COVID-19
Panic buying pushes up prices of masks, hand sanitiser (12)	Mar 9 2020	The Daily Observer	Discussed an panic buying episode	Detection of COVID-19 in Bangladesh	Local	Masks, toilet paper, hand sanitizer	Increase demand and price		COVID-19
Yarn prices soar as virus fear triggers panic buying (13)	Mar 10 2020	The Daily Star	It describes the effect of PB on industry buying increasing the price of Yarn	COVID-19 pandemic	Local	Yarn	Increase demand and price, stock out		COVID-19
Virus outbreak fears spark panic buying in Bangladeshi capital (14)	Mar 11 2020	Arab News	It reported the PB event and prevention activity	Uncertinity due to COVID-19 pandemic	International	Masks, sanitizers	Increase demand, stock out	Rationing	COVID-19
City people go for panic buying, govt says there's ample supply (15)	Mar 17 2020	Newage	It reported the PB event and prevention activity	COVID-19 pandemic	Local	Rice, rice, flour, lentil, pulse, oil, salt, sugar, hand-wash, sanitizers	Increase demand, increased price, people buying extra amount	Assurance of stocks from the Government, Rationing from the super shops	COVID-19
Minister's assurance ignored, panic buying spree continues (16)	Mar 18 2020	The Business Statndard	The report discussed the Government's initiatives to stop PB	COVID-19 pandemic	Local	Rice, lentil, sugar, milk powder	Increase demand, increased price, people buying extra amount	Raising awareness, selling goods in lower price by the Govt., formulation of monitoring, punishment to maleficent sellers, dissemination of stock status to the general people	COVID-19
First coronavirus death news prompts panic buying (17)	Mar 18 2020	The Business Standard	It reported the PB event and prevention activity	First death due to COVID-19	Local	Soybean oil, chicken, fish, milk powder, rice	Increase demand, increased price, people buying extra amount	Raising awareness, formulation of special team to monitor, punishment to maleficent sellers, dissemination of stock status to the general people	COVID-19
Panic buying pushes commodity prices up (18)	Mar 19 2020	Financial Express	The report discussed the PB episode and government's action to prevent it	COVID-19 pandemic	Local	Rice, pulses, flour, potato, beef, egg, spices, onion, garlic, ginger, biscuits, baby diaper, toilet paper and mosquito coils	Increase demand, increased price, people buying extra amount	Raising awareness, selling goods in lower price by the Govt., formulation of monitoring team, punishment to maleficent sellers, dissemination of stock status to the general people	COVID-19
Coronavirus triggers panic buying in Dhaka as stocks drop to 7-year low, gold prices fall (19)	Mar 19 2020	BDNews24.com	The report discussed the PB episode and government's action to prevent the PB	COVID-19 pandemic	Local	Rice, lentil	Increase demand, stock out	Raising awareness, dissemination of stock status to the general people	COVID-19
Panic buying on amid coronavirus fear in Bangladesh (20)	Mar 20 2020	Newage	It reported a PB event and the Government's action to control	First death due to COVID-19	Local	Rice, onion, garlic, lentil, potato, masks, hand sanitizers, hand rubs, soaps, hygiene products, noodles, oil, sugar, salt	Increase demand, increased price, people buying extra amount	Formulation of monitoring team, punishment to maleficent sellers, assurance of stocks	COVID-19
Online sales jump on panic buying	Mar 20 2020	The Finance Today	It describes the increased orders in ecommerce business	anticipation of an impending lockdown due to COVID-19	Local	rice, lentil, sugar, hand sanitizers	Increase demand		COVID-19
Onion prices double due to coronavirus panic-buying (21)	Mar 20 2020	Jagonews	It discussed an panic buying episode	COVID-19 pandemic	Local	Onion, ginger, garlic, potato			COVID-19
Coronavirus: Panic buying doubles onion prices in Dhaka (22)	Mar 21 2020	UNB NEWS	It described an episode of PB	COVID-19 lockdown	Local	Onion, garlic, rice, potato, egg, soybean oil, lentil	Increase demand, increased price, people buying extra amount	Mentioned the Government's action to control it	COVID-19
No panic buying, says PM (23)	Mar 21 2020	Somoy News	The report discussed the prevention steps of the Government for PB	COVID-19 pandemic	Local	Rice, pulse, egg and onion		Raising awareness, assurance about the stock	COVID-19
Online shopping gets momentum in BD for Corona panic (24)	Mar 21 2020	Daily Industry	The report discussed the effects of PB on online markets	COVID-19 pandemic	Local	rice, lentil, sugar	Increase demand		COVID-19
Panic buying prompts large jump in rice price (25)	Mar 23 2020	ProthomAlo	It reported the PB event of rice and recommended Governmental action to control	anticipation of an impending lockdown due to COVID-19	Local	Rice, Onion, garlic, potato, Soybean, lentil, eggs	Increase demand, increased price, people buying extra amount		COVID-19
Panic buying of medicines, self-prescription on rise (26)	Mar 25 2020	Bangladesh Post	It reported the PB event of medications and expert opinion	COVID-19 pandemic	Local	Antibiotics, cough syrup and anti-cold pills, fever medicine, Alatrol, Histasin, Paracetamol and C vitamins	Increase demand	Raising awareness	COVID-19
Covid-19: Panic buying drives pulse oximeter price up (27)	Jun 16 2020	DhakaTribune	It described a PB episode	COVID-19 pandemic	Local	Pulse oximeter	Increase demand, increased price		COVID-19
Panic-buying creates shortage of “Covid-19 drugs” in the market (28)	Jun 21 2020	DhakaTribune	It described a PB episode of medications, their side effects, and Government's action	COVID-19 pandemic	Local	Hydroxychloroquine tablets, dexamethasone, azithromycin, ivermectin, paracetamol, montelukast, doxofylline, salbutamol, fexofenadine, and some vitamin tablets	Increase demand	The Government published circulars in newspapers to raise awareness	COVID-19
Cease panic buying, enough in supply (29)	Sep 17 2020	NewsToday	The report discussed the prevention steps of the Government of a PB episode	export ban of Onion of India	Local	Onion		Import from alternative sources, reduced use of onion	Export ban
Tipu: Dishonest traders, panic buying behind onion price hike (30)	September 17 2020	Business outlook	It discussed the prevention steps of the Government of a PB episode of onion	export ban of Onion of India	Local	Onion	Increase demand, increased price	Raising awareness, selling goods in lower price by the Government, dissemination of stock, supply and price situation to the general people, reduction of import duty	Export ban

### Responsible Factors

Several attributing factors were identified from the reports such as rumor of danger (flood), curfew, policy ban, uncertainty, the anticipation of an impending lockdown, increased demand, anticipation of price hike, and anticipation of short supply (Table 1).

### Goods of Panic Buying

The reported goods are necessary for daily living such as rice, sugar, salt, onion, pulse, potatoes, spices, masks, sanitizers, toilet paper, flour, lentil, pulse, oil, milk powder, chicken, fish, beef, egg, garlic, ginger, biscuits, baby diaper, mosquito coils, soaps, hygiene products, noodles, drugs (antibiotics, cough syrup, and anti-cold pills, fever medicine, hydroxychloroquine, dexamethasone, ivermectin, montelukast, doxofylline, salbutamol, some vitamin tablets), and pulse oximeter (Table 1).

### Prevention Strategies

Media reports frequently mentioned prevention strategies, expert opinion, supply chain status, rationing, and government action. Raising awareness, selling goods at a lower price by the government, formulation of the special monitoring team, punishment to maleficent sellers, dissemination of stock status to the general people, assurance of stocks, import from alternative sources, reduced use of goods (onion), rationing while selling from the super shops, publishing circulars in newspapers to raise awareness, and a reduction of import duty were the controlling measures identified by the analysis.

## Discussion

Panic buying is an under-researched area even though it is common behavior during emergencies. This study aimed to identify the characteristics of panic buying episodes in Bangladesh in comparison to current concepts. We checked 24 published news reports (Table 1) to identify the precipitating events, products, events, responsible factors, goods of panic buying, and prevention measures. Five panic buying episodes were identified.

### Current Concepts of PB

From the available evidence, we established that PB usually starts with an adverse stimulus, and people usually buy necessary goods in response to several psychological reactions (1–4). Media has a bidirectional role on PB which can either control or exacerbate (6, 7). Controlling media reporting, kinship promotion, the rationing of the products, assurance regarding safe supply, policy change, raising awareness, selling goods at a subsidized price have been noted as prevention strategies (6, 7).

### Main Findings of the Study

The first episode happened in 1998 where a flood acted as a precipitating event. During this event rice, wheat, sugar, salt, onion, pulse, potatoes, and spices were brought, and assurance of adequate stocks was disseminated (8). The second episode happened in 2007 where curfew due to student riot acted as a precipitating event and necessary goods were brought (9). The third episode happened in 2019 where the export ban of the onion of a neighboring country acted as a precipitating event (10). The fourth episode was precipitated by the COVID-19 pandemic (11–29). The fifth episode was precipitated by another export ban of the onion by India (30, 31). Media reports frequently mentioned prevention strategies, expert opinion, supply chain status, rationing, and Government action (Table 1). The reported goods are necessary for daily living, for example, medications and safety products (Table 1). Several controlling measures were practiced in Bangladesh during the episodes and the government used initiatives. The episodes describing the characteristics of PB fit with the existing concept of panic buying in regards to precipitating events, responsible factors, goods, and measures. Although the episodes covered two different times (COVID-19 and others) the characteristics are similar.

The study revealed primitive characteristics of panic buying episodes, where it starts with adverse stimuli, have an extra buying of necessary goods, resulting in disruption of supply chains supporting the existing causative model of PB proposed by Arafat et al. (1). The practiced prevention strategies have also been supported by previous recommendations (31). Other studies also reported on items sought panic buying which are usually daily necessities (4, 32). The media reports have positive reporting characteristics, as revealed by recent studies (6).

### Strengths of the Study

This is the first systematic assessment of panic buying in Bangladesh and reveals preliminary explorative findings that could help to formulate prevention strategies in future.

### Limitations

Data were extracted from media reports which are not of scientific quality. The sample size was small. Data extraction and the search were done by a single person (first author). No structured instrument was used to extract the data. Only English language reports were studied.

## Conclusion

The study revealed preliminary findings of panic buying in Bangladesh. However, they are aligned with the current concept of panic buying. Further empirical studies are warranted to explore geographical variation, demographics (e.g., education, income), consumption behavior, precise factors, and to test culturally appropriate control measures.

## Data Availability Statement

The raw data supporting the conclusions of this article will be made available by the authors, without undue reservation.

## Ethics Statement

The study was conducted complying with the declaration of Helsinki (1964). As we analyzed the publicly available media reports, no formal ethical approval was obtained.

## Author Contributions

SA contributed to the concept, design, data analysis, and writing. All authors contributed to manuscript writing, revision, read, and approved the submitted version.

## Conflict of Interest

The authors declare that the research was conducted in the absence of any commercial or financial relationships that could be construed as a potential conflict of interest.
